# Bioaccumulation of Trace Elements from Aqueous Solutions by Selected Terrestrial Moss Species

**DOI:** 10.3390/biology11121692

**Published:** 2022-11-23

**Authors:** Paweł Świsłowski, Arkadiusz Nowak, Stanisław Wacławek, Daniele Silvestri, Małgorzata Rajfur

**Affiliations:** 1 Institute of Biology, University of Opole, 45-032 Opole, Poland; 2 Polish Academy of Sciences, Botanical Garden—Centre of Biodiversity Conservation, 02-973 Warsaw, Poland; 3 Department of Botany and Nature Protection, University of Warmia and Mazury in Olsztyn, 10-721 Olsztyn, Poland; 4 Institute for Nanomaterials, Advanced Technologies and Innovation, Technical University of Liberec, 461 17 Liberec, Czech Republic

**Keywords:** bioaccumulation, sorption, mosses, heavy metals, FTIR, urban areas

## Abstract

**Simple Summary:**

In this study, the kinetics of elemental sorption in moss species were investigated under laboratory conditions. Functional groups responsible for binding metal cations to the internal structures of the mosses were also identified. Based on the conducted research, it was found that regardless of the moss species, a state of equilibrium in the moss–solution system was reached after 60 min, as indicated by the stable readings of measuring instruments. Under the conditions of the experiment, in the first 10 min of the process about 70.4–95.3% of metal ions were sorbed from the solution into the moss gametophytes with respect to the concentration of this analyte accumulated in the mosses at equilibrium. The results of the study indicate that the process of bioaccumulation of heavy metals in mosses occurs mainly through ion exchange, as evidenced—among other things—by a decrease in the concentration of metal ions in the solution with which they are in contact and a concomitant increase in the conductivity in a solution. The presented results indicate the interrelationship between concentrations of cations in and around mosses (solution/atmospheric aerosols). At the same time, the presented results make it possible to identify and select appropriate moss species for biomonitoring purposes.

**Abstract:**

The interrelationship between metal concentrations in mosses and their surroundings prompts research toward examining their accumulation properties, as it is particularly important for their usage in biomonitoring studies that use mosses. In this study, the kinetics of elemental sorption in three moss species (*Pleurozium schreberi*, *Dicranum polysetum*, and *Sphagnum fallax*) were investigated under laboratory conditions. Sorption from metal salt solutions was carried out under static conditions with decreasing elemental concentration. Functional groups responsible for binding metal cations to the internal structures of the mosses were also identified. It was shown that the equilibrium state was reached after about 60 min. Under the conditions of the experiment, in the first 10 min of the process, about 70.4–95.3% of metal ions were sorbed from the solution into the moss gametophytes by *P. schreberi* (57.1–89.0% by *D. polysetum* and 54.1–84.5% by *S. fallax*) with respect to the concentration of this analyte accumulated in the mosses at equilibrium. It can be assumed that the exposure of mosses with little contamination by heavy metals in an urbanized area under active biomonitoring will cause an increase in the concentration of these analytes in proportion to their concentration in atmospheric aerosols. In the case of *P. schreberi* and *D. polysetum*, the O-H/N-H band was enormously affected by the adsorption process. On the other hand, FTIR (Fourier transform infrared spectroscopy) analysis of *S. fallax* after adsorption showed slight changes for most of the bands analyzed. Based on this study, it can be concluded that mosses can be used as, for example, a biomonitor in monitoring of urban ecosystems, but also in the phytoremediation of surface waters.

## 1. Introduction

Over the past 50 years, studies of the application of mosses and lichens in biomonitoring have grown significantly and have either expanded or should be still expanded in sampling techniques [1,2]. With the continuous systematization and improvement in research methodologies, biomonitoring with mosses and lichens continues to find new opportunities for their application in environmental quality monitoring. Recent examples include magnetic biomonitoring with lichens in assessing the impact of dust pollution on cultural heritage [3] or the mobile air quality monitoring capabilities of the moss-bag technique [4]. In this research, it is important to determine whether we are studying bioconcentration, surface adsorption of trace elements, or both. [5].

Most biomonitoring studies emphasize the high percentage of contaminants and particle deposition on mosses’ surfaces [6,7]. The different sizes of these particles and their ability to adhere to biological material is strongly related to the structure of the leaf. The fact that the more particles are deposited on the moss leaves the higher the concentration of trace elements is confirms that their uptake by mosses is mainly based on passive mechanisms (depending on the properties of the moss surface) [8,9]. Only a small percentage of their total concentration is accumulated inside tissues, either in soluble form or bound to the inner plasma membrane [10]. On one hand, there is evidence that washing samples has proven to be ineffective for the determination of the bioaccumulated fraction in terrestrial mosses [11]. On the other hand, we have to consider that washing samples is necessary for determining the polycyclic aromatic hydrocarbon (PAH) bioconcentration in mosses [12]. The use of various analytical and laboratory techniques makes it possible to assess the bioconcentration of compounds and trace elements and their impact on the viability of biomonitors [13,14,15]. Despite the many advantages of using clones and devitalized biomonitors [16,17], we stand by our position of measuring and controlling the vital parameters of mosses during biomonitoring studies [18,19] in order to determine the real response of a living organism to environmental pollution and not just that of dead tissue as a chemical adsorbent [20,21]. In environments with high levels of atmospheric heavy metal deposition, the photosynthesis of *Pseudoscleropodium purum* is inhibited and protein expression in this moss is susceptible to modifications related to environmental conditions [22]. Other studies have ruled out the possibility of using the moss *Ptychostomum capillare* in biomonitoring of air pollution due to a defense mechanism mediated by lipophilic substances (probably waxes) that act as a barrier to prevent metal from entering the cells, which could distort direct estimates of environmental copper content through levels detected in its tissues [23]. Thus, understanding the effects of analytes on bryophyte morphophysiological traits may be fundamental to optimizing their use in biomonitoring [24]. Therefore, in addition to just studying the surface sorption of trace elements by mosses, it is necessary to focus on the bioconcentration of these elements in their interior, and look for relationships between particles attached to the surface and those bioaccumulated in their tissues [12].

The aim of this study is to determine whether a variation in the bioconcentrations of selected metals in mosses of three species: *P. schreberi*, *S. fallax*, and *D. polysetum* accumulated from aqueous solutions of these elements. The sorption (physical and chemical process [absorption, adsorption, ion exchange] by which a metal’s cations accumulated in the mosses) of five anthropogenic-derived metals that are often found in atmospheric aerosols was analyzed. We have tried to verify the research hypothesis that mosses actively accumulate analytes into their tissues during exposure to pollutants. We expect to provide evidence supporting this hypothesis by several means: (1) analysis of heavy metal ion sorption kinetics in moss gametophytes, and (2) identification of the main functional groups present in the tissues of mosses that are responsible for the accumulation of metals.

## 2. Materials and Methods

The species used for this study were the moss *P. schreberi* (Pl), *S. fallax* (Sp), and *D. polysetum* (Dp). They were collected in October 2021 from forests in the Swietokrzyskie Voivodship in southeastern Poland.

Moss samples were taken and treated before exposure as part of active biomonitoring in accordance with standard guidelines [25]. The mosses were prepared before exposure according to a previously developed methodology [26]. The concentrations of metals naturally accumulated in the mosses used for the experiments [mg/g dry weight] are presented in the Supplementary Materials (Table S1).

This research was conducted on whole, live/fresh (quantum yield of chlorophyll fluorescence was 0.5–0.7, see below) moss gametophytes of *P. schreberi*, *S. fallax*, and *D. polysetum*. Moss samples weighing 0.200 ± 0.001 g were placed in a perforated container with a volume of about 15 cm^3^ and immersed in a salt solution of the selected metal with a volume of 200 cm^3^. The solution was stirred using a magnetic stirrer (VELP Scientifica Srl, Usmate, IT; RPM: 250). Periodically, the solution was drawn directly from the vessel in which the experiment was conducted to determine (AAS) the concentration of the metal. Metal salts were dissolved in deionized water. The uncertainty of the measurements of metal concentrations using AAS did not exceed 5%. The process was carried out for about 60 min, as previous studies have shown that a state of equilibrium in the moss–solution system (stable readings of measuring instruments) is achieved within this time [27]. The concentrations of analytes were chosen so that it would be possible to follow the kinetics of the process using F-AAS and also so that the concentrations would be comparable to those found in the environment. During the process of metal accumulation in moss gametophytes the measurement of changes in the conductivity and pH of the solution were also conducted. After the process, the mosses were rinsed with deionized water and left for 60 min under laboratory conditions, and then their viability (measure of how much plant material in a lot are alive) and actual photochemical efficiency (yield) were measured. The actual quantum efficiency of photosystem II (PSII) photochemistry in the light measures the fraction of the absorbed light energy that is actually being used to drive photochemistry at PSII [28]. Estimates of the efficiency at which light absorbed by PSII are used for photochemistry; at a given light intensity, it provides an estimate of the quantum efficiency of linear electron transport through PSII [29]. The samples were dried at room temperature and were then destined for Fourier transform infrared spectroscopy (FTIR) analysis to study the moss gametophytes before and after the metal accumulation process (c_Mt_,_0_ = 2.66 mg/L—initial concentration of metal salt solutions to which the mosses were introduced). For this purpose, 0.200 ± 0.001 g of moss was dried at room temperature and then homogenized using a mortar.

### 2.1. Devices and Reagents

Heavy metals were determined using an iCE 3500 atomic absorption spectrometer from Thermo Electron Corporation (Grand Island, NY, USA). A CP551 pH meter (Elmetron Sp. j. Zabrze, PL) and CC551 conductivity meter (Elmetron Sp. j. Zabrze, PL) were used, whose absolute error of indication was ΔpH = 0.02 and Δκ = 0.1 μS/cm, respectively, to test the conductivity and pH of the solutions in which the mosses were immersed. Solutions of metal salts of Ni, Cu, Zn, Cd, and Pb were prepared using reagents from MERCK. The uncertainty in the readings of the laboratory balance used was ± 0.001 g. For identification of the potential functional groups and possible binding sites associated with the accumulation of Ni(II), Cu(II), Zn(II), Cd(II), and Pb(II), IR analysis was performed using an FTIR spectrometer (Fourier transform infrared spectrometer, Cary 630 FTIR spectrometer, NICOLET IZ10, Thermo Scientific, Waltham, MA, USA).

The chlorophyll fluorescence of PSII was monitored using a modulated portable fluorometer (Opti-Sciences, Hudson, NH, USA). Actual photochemical efficiency (yield) was measured under ambient light [30]. Actual photochemical efficiency (yield) below 0.1 is the critical value below which the moss is only a natural sorbent and not a biomonitor because it loses its viability [31].

### 2.2. Quality Control

Table 1 shows the limits of detection and limits of quantification of heavy metals characterizing the iCE 3500 spectrometer [32]. Values of the highest concentrations of standards (ANALYTIKA Ltd., Prague, Czech Republic) used for calibration (2.0 mg/dm^3^ for Cd, 5.0 mg/dm^3^ for Cu, Zn, Ni, and Pb) were taken as the limit of the linear dependence of the signal on concentration.

Table 2 shows the concentrations of trace elements determined in the certified reference material BCR-482 *lichen*, produced by the Institute for Reference Materials and Measurements, Belgium.

## 3. Results and Discussion

The use of several moss species at once is crucial for monitoring elemental deposition in the environment due to the possibility of comparing their accumulation capacities [33]. Studies of the kinetics of the metal ion accumulation process were carried out under static conditions, with continuous stirring of the solution. In the first stage of the study, the concentrations of selected metals were evaluated in solutions of their salts into which mosses were immersed. The process was followed using the AAS technique until an equilibrium state (condition resulting from a heterophasic double exchange reaction between mobile cations bound in the cell wall of mosses and the composition of the solution with which the mosses come into contact) was reached between the mosses and the solution in which they were immersed. For this purpose, samples of mosses were immersed in salt solutions of selected metals with a volume of 200 cm^3^ and a specified initial concentration of metal in solution c_s,0_. Table 3 shows the initial concentrations of metals c_s,0_, the concentrations of trace elements after the sorption process in solution c_s,1_, and accumulated in mosses at equilibrium state c_M,1_. The concentrations of sorbed metals per unit mass of mosses were determined from the relationship

(1) $$c_{M,1}=\frac{\left(c_{s,0}-c_{s,1}\right)*V}{m}$$

where *V* = the volume of solution from which sorption was carried out and *m* = the mass of the moss.

From these experiments, aqueous solutions of analytes with concentrations of about 2.60–2.80 mg/dm^3^ were used for the second stage of the study, which aimed to follow the kinetics of the sorption process in the moss–solution system using AAS. The use of solutions with such concentrations of individual metals made it possible to run the tests until equilibrium was reached in the moss–aqueous solution system; at the same time, the concentrations of analytes were close to those found in the environment [34]. Mosses, due to their specific anatomical structure and specific mode of nutrition, sorb nutrients contained in atmospheric aerosols [35]. The process of trace element accumulation occurs mainly from aqueous solutions; as the concentration of an analyte in solution increases, its concentration in moss gametophytes increases [36], as shown in Table 3.

**Figure 1:**
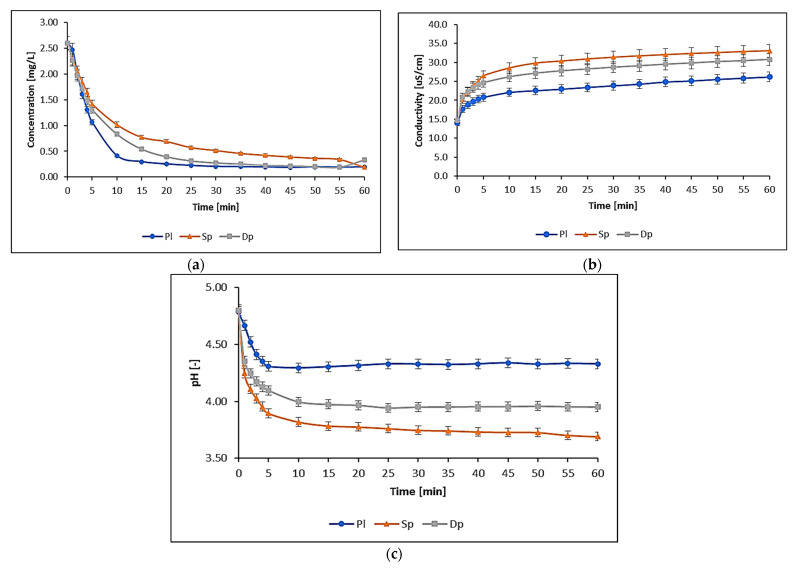
Changes in physicochemical parameters in Cu solution during the accumulation process of moss gametophytes: (**a**) concentration; (**b**) conductivity; (**c**) pH (Pl—*P. schreberi*, Sp—*S. fallax*, Dp—*D. polysetum*). Whiskers indicate standard deviation levels.

Figure 1 shows the changes in elemental concentration, conductivity, and pH in salt solutions of the analyzed metals after immersing selected moss species in them and conducting the sorption process.

It can be concluded from the experiments that regardless of the moss species, in the moss–solution system a state of equilibrium was reached after 60 min, as indicated by stable readings of conductivity and pH of the solution and the absence of significant changes in the concentrations of trace elements in the solutions (see Table 3 and Figure 1a). A slight increase in the copper (60 min) concentration in solution with *D. polysetum* could be due, for example, to the dissolution of salts/dust naturally accumulated on the moss. Subsequent measurements of concentrations were stable. In comparison, the state of dynamic equilibrium during the process of sorption of Cu^2+^ ions in the marine algae *Palmaria palmata* was reached after about 70 min, and in the freshwater algae *Spirogyra* sp. after about 30 min [37]. In the moss *P. schreberi*, 80.5–97.0 % of the metal ions found in the initial solution (in *D. polysetym*—74.2–96.3%; in *S. fallax*—81.6–95.2%) accumulated during the 60 min process (Table 3). In the first 10 min of the process, about 70.4–95.3% of metal ions were sorbed from the solution to the moss gametophytes by *P. schreberi* (57.1–89.0% by *D. polysetum*; 54.1–84.5% by *S. fallax*) relative to the concentration of this metal accumulated in the mosses at equilibrium (Supplementary Materials—Figures S1–S4). The intensity of analyte accumulation in mosses depends, among other things, on their affinity for the functional groups that make up the compounds that form the cell wall, as well as the structure and development of the surface of the moss gametophyte [38]. Under the conditions of the research conducted, the accumulation of trace elements depended on the species of moss, increasing in a series: *S. fallax* < *D. polysetum* < *P. schreberi*. Evaluation of sorption properties is important when using different moss species in biomonitoring studies.

**Figure 2:**
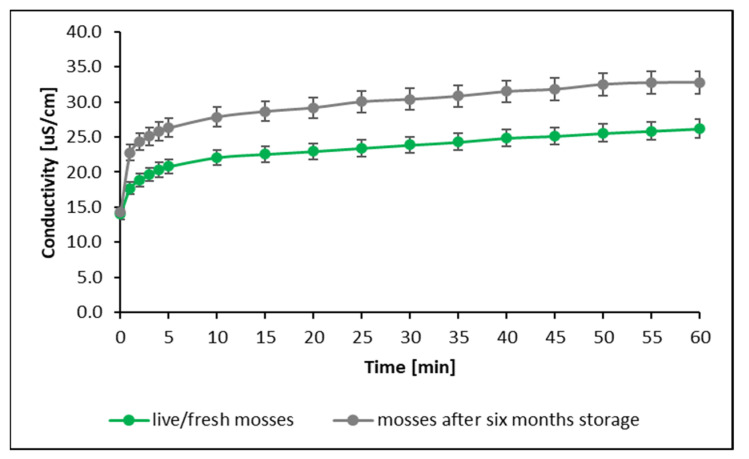
Conductivity changes during the process of Cu sorption on the moss *P. schreberi* (mosses stored in the laboratory for 6 months were used for the study).

Figure 1c shows that regardless of species, the process of Cu sorption by mosses is accompanied by the sorption of H^+^ ions. An increase in conductivity was also spotted during the process (Figure 1b). Figures of physicochemical changes for the remaining trace elements are presented in the Supplementary Materials (Figures S1–S4). The increase in the solution’s conductivity after the insertion of mosses into the solution was caused, among other things, by the dissolution of salts naturally accumulated on the surface of the mosses and progressive, irreversible changes in the structure of cell membranes over time, which cause the leakage of ionic substances from moss cells to the solution [39,40,41]. This process can be observed using conductivity measurements to track the kinetics of metal sorption on mosses that have been stored for 6 months (Figure 2).

The changes in the structures of cell membranes, occurring over time and causing an increase in the amount of ions in solution, were observed through an increase in conductivity as well as a decrease in the pH of the solution (an increase in the concentration of hydrogen ions), which also led to a reduction in Cu sorption on moss gametophytes by about 5% (under experimental conditions, regardless of moss species). At the same time, the reduction in the vitality of mosses after a period of 6 months was confirmed by studies using the actual photochemical efficiency of PSII. Live/fresh mosses were characterized by a vitality of 0.6–0.7, while for mosses stored for a period of 6 months the vitality dropped to 0.1 (anabiosis state) but they did not lose their accumulative properties [20]. Various devitalized matrices (mosses, lichens) are commonly used in the literature because of the ease of working with such material, as well as precisely because of their lack of loss of uptake ability [18,42].

Literature data indicate that the process of heavy metal accumulation in mosses occurs mainly through ion exchange and is surface-based [43]. The same effect was observed during the study of heavy metal sorption processes in lichens [44]. However, it should be noted that in parallel or secondarily, metals accumulate in the intracellular structures of mosses [38,45].

**Figure 3:**
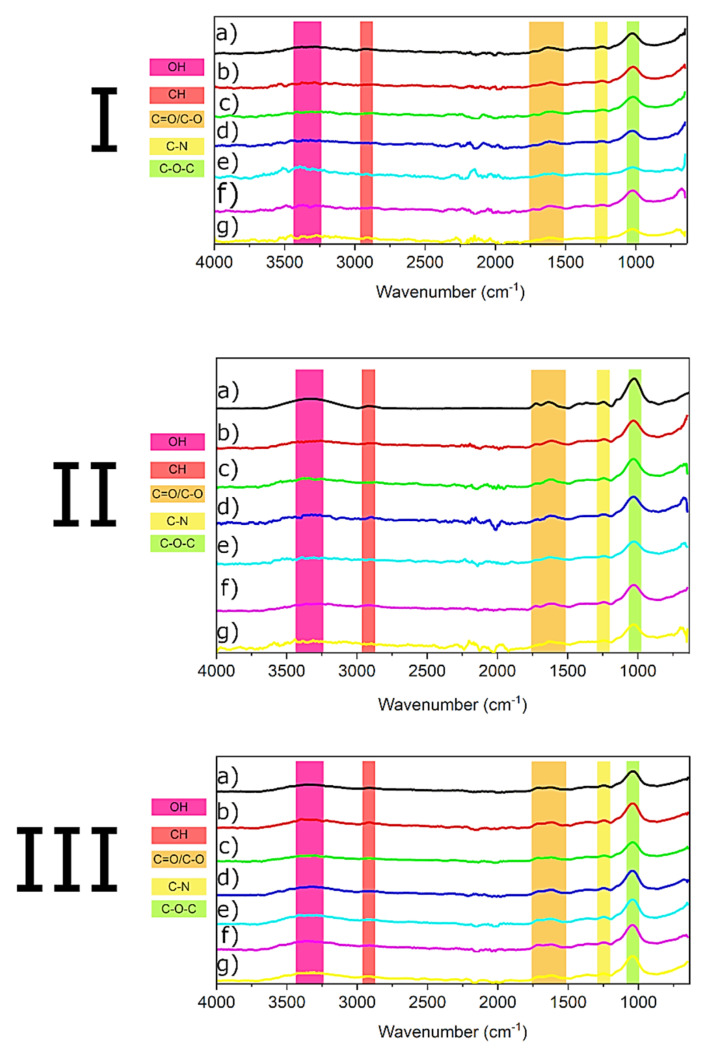
FTIR of (**I**) *P. schreber*i (**a**) before adsorption, (**b**) after Ni^2+^ adsorption, (**c**) after Cu^2+^ adsorption, (**d**) after Zn^2+^ adsorption, (**e**) after Cd^2+^ adsorption, (**f**) after Pb^2+^ adsorption, and (**g**) mixture of cations. (**II**) *D. polysetum* (**a**) before adsorption, (**b**) after Ni^2+^ adsorption, (**c**) after Cu^2+^ adsorption, (**d**) after Zn^2+^ adsorption, (**e**) after Cd^2+^ adsorption, (**f**) after Pb^2+^ adsorption, and g) mixture of cations. (**III**) *S. fallax* (**a**) before adsorption, (**b**) after Ni^2+^ adsorption, (**c**) after Cu^2+^ adsorption, (**d**) after Zn^2+^ adsorption, (**e**) after Cd^2+^ adsorption, (**f**) after Pb^2+^ adsorption, and (**g**) mixture of cations.

FTIR analysis of the main functional groups found in the mosses that are responsible for metal accumulation was carried out (Figure 3).

An FTIR analysis was carried out to better understand the adsorption mechanisms involved in removing metal cations. Therefore, mosses before adsorption and after the removal of metals were compared (Figure 3). Mosses have been reported to be composed mainly of carbohydrates (>50%), lipids, and proteins [46,47], which were all identified by FTIR spectra in *P. schreberi, D. polysetum*, and *S. fallax*. Overall, the mosses’ spectra have various regions of interest. The first one (~3700–~3000 cm^−1^) represents O-H and N-H bonds [48] (symmetric and asymmetric stretch) from carbohydrates and proteins. Following peaks could be observed at ~2913 cm^−1^ C-H (stretch) from polysaccharides, lipids, and carbohydrates, while at ~1716 and ~1616 cm^−1^ the peaks were ascribed respectively to C=O [49] and N-H [50]. At ~1243 cm^−1^ another peak appeared that could be attributed to the presence of amide (C-N stretch) [51] from proteins and glycoproteins. Then, at ~1040 cm^−1^_,_ we could identify the signal of C-O-C functionalities [52] (stretch) from oligosaccharides, glycoprotein, and carbohydrates.

As Figure 3 shows, after adsorption with various metal cations, several peaks changed in intensity compared to the spectra of mosses before adsorption. In the case of *P. schreberi* and *D. polysetum*, the O-H/N-H band is tremendously affected after the adsorption process. In the same way, the bands at ~1716 and ~1616 cm^−1^ underwent changes. Finally, the last two bands at ~1250 and ~1040 cm^−1^ also show an intensity reduction but to a lesser extent than the previous one. On the other hand, FTIR of *S. fallax* after adsorption showed slight changes for most of the bands analyzed. In Figure 3 III, the dominant changes are in the region of ~3700–~3000 cm^−1^. The adsorption of metals in mosses could be due to electrostatic interactions between the ions and negatively charged functional groups on them, as reported previously by Vinod and Sashidhar [53]. The apparent decrease in intensity of the O-H/N-H peak could indicate that the bonded O-H functional groups and amide moieties may play an essential part in the sorption of metal ions. A similar observation was reported for the adsorption of Ni^2+^ by a carbohydrate gum adsorbent [54]. In addition, a decrease in intensity of the ~1716 and ~1616 cm^−1^ peaks after adsorption could indicate participation of the C=O and N-H groups in conjugating the metal cations.

As reported by González and Pokrovsky [55], metal cations can bind to the surface layers of the cell wall via cation exchange. Several substances make up the cell walls of mosses, such as cellulose and glycoproteins, which can be involved in the adsorption of metals, as reported by Nag [56] and Maruyama [57], respectively. Therefore, based on FTIR analysis and the literature [55], it seems likely that the surface layers of the mosses are responsible for metal adsorption. In addition, the slightly different mosses’ cell wall compositions can influence metal adsorption [58].

## 4. Conclusions

The results of this study indicate that the process of bioaccumulation of heavy metals in mosses occurs mainly through ion exchange as evidenced, among other things, by a decrease in the concentration of metal ions in the solution with which they are in contact and a concomitant increase in the conductivity of a solution. Based on the conducted research, it was found that regardless of the moss species, in the moss–solution system, a state of equilibrium was reached after 60 min, as indicated by stable readings of conductivity and pH of the solution and the absence of significant changes in the concentrations of trace elements in the solution. It should be noted, however, that in parallel or secondarily, metals accumulate in the intracellular structures of mosses. FTIR analysis of moss samples confirmed the participation of hydroxy, amine, and carbonyl groups in the biosorption process of metal cations.

The presented results of this study indicate the interrelationship between the concentration of cations in and around mosses (solution/atmospheric aerosols). At the same time, the presented results make it possible to identify and select appropriate moss species for biomonitoring purposes.

## Figures and Tables

**Table 1:** The instrumental detection limits (*IDL*) and instrumental quantification limits (*IQL*) for the iCE 3500 spectrometer [mg/L] [32].

Metal	*IDL*	*IQL*
Ni	0.0043	0.050
Cu	0.0045	0.033
Zn	0.0033	0.010
Cd	0.0028	0.013
Pb	0.0130	0.070

**Table 2:** Comparison of measured and certified concentrations in BCR-482 *lichen*.

	BCR-482 *lichen* (Certified)		AAS (Measured)		*Dev*.**
Metal	Concentration	±Uncertainty	Mean	±*SD* *	
	[mg/kg dry weight]				[%]
Ni	2.47	0.07	2.16	0.32	−13
Cu	7.03	0.19	6.63	0.17	−5.7
Zn	100.6	2.2	95.1	2.3	−5.5
Cd	0.56	0.02	0.53	0.03	−5.3
Pb	40.9	1.4	38.2	1.0	−6.6

Note: * standard deviation. ** relative difference between the measured (c_m_) and certified (c_c_) concentration 100% (c_m_—c_c_)/c_c_.

**Table 3:** Changes in elemental concentrations in solutions [mg/L] and in mosses [mg/g d.w.] during the sorption process (*n* = 3).

*Pleurozium schreberi*			*Dicranum polysetum*			*Sphagnum fallax*		
***c*_s,Ni(0)_**	***c*_s,Ni(1)_**	***c*_M,1_**	***c*_s,Ni(0)_**	***c*_s,Ni(1)_**	***c*_M,1_**	*c*_s,Ni(0)_	*c*_s,Ni(1)_	*c*_M,1_
0.07	<0.05	>0.02	0.07	<0.05	>0.02	0.07	<0.05	>0.02
0.30	<0.05	>0.25	0.30	<0.05	>0.25	0.30	<0.05	>0.25
2.67	0.52	2.15	2.67	0.69	1.98	2.67	0.49	2.18
*c*_s,Cu(0)_	*c*_s,Cu(1)_	*c*_M,1_	*c*_s,Cu(0)_	*c*_s,Cu(1)_	*c*_M,1_	*c*_s,Cu(0)_	*c*_s,Cu(1)_	*c*_M,1_
0.05	<0.03	>0.02	0.05	<0.03	>0.02	0.05	<0.03	>0.02
0.30	<0.03	>0.27	0.30	<0.03	>0.27	0.30	<0.03	>0.27
2.60	0.20	2.40	2.60	0.19	2.40	2.60	0.33	2.30
*c*_s,Zn(0)_	*c*_s,Zn(1)_	*c*_M,1_	*c*_s,Zn(0)_	*c*_s, Zn (1)_	*c*_M,1_	*c*_s, Zn (0)_	*c*_s, Zn (1)_	*c*_M,1_
0.03	<0.01	>0.20	0.03	<0.01	>0.02	0.03	<0.01	>0.02
0.30	0.02	0.28	0.30	0.02	0.28	0.30	0.01	0.29
2.81	0.40	2.41	2.81	0.64	2.17	2.81	0.49	2.32
*c*_s,Cd(0)_	*c*_s, Cd (1)_	*c*_M,1_	*c*_s, Cd (0)_	*c*_s, Cd (1)_	*c*_M,1_	*c*_s, Cd (0)_	*c*_s, Cd (1)_	*c*_M,1_
0.03	<0.01	>0.02	0.03	<0.01	>0.02	0.03	<0.01	>0.02
0.30	<0.01	>0.29	0.30	<0.01	>0.29	0.30	0.07	0.23
2.67	0.09	2.58	2.67	0.27	2.40	2.67	0.23	2.44
*c*_s,Pb(0)_	*c*_s, Pb (1)_	*c*_M,1_	*c*_s, Pb (0)_	*c*_s, Pb (1)_	*c*_M,1_	*c*_s, Pb (0)_	*c*_s, Pb (1)_	*c*_M, 1_
0.09	<0.07	>0.02	0.09	<0.07	>0.02	0.09	<0.07	>0.02
0.24	<0.07	>0.17	0.24	<0.07	>0.17	0.24	<0.07	>0.17
2.70	0.08	2.62	2.70	0.10	2.60	2.70	0.13	2.57

## Data Availability

Not applicable.

## Supplementary Materials

The following supporting information can be downloaded at: https://www.mdpi.com/article/10.3390/biology11121692/s1, Figure S1: Physicochemical changes in Ni solution during the accumulation process on moss gametophytes: (a) concentration, (b) conductivity, (c) pH. Figure S2: Physicochemical changes in Zn solution during the accumulation process on moss gametophytes: (a) concentration, (b) conductivity, (c) pH. Figure S3: Physicochemical changes in Cd solution during the accumulation process on moss gametophytes: (a) concentration, (b) conductivity, (c) pH. Figure S4: Physicochemical changes in Pb solution during the accumulation process on moss gametophytes: (a) concentration, (b) conductivity, (c) pH. Table S1: Concentrations of metals naturally accumulated in the mosses used for the experiments [mg/g d.w.].
