# Supplementary material for: Bioaccumulation of Trace Elements from Aqueous Solutions by Selected Terrestrial Moss Species
Source: Biology (Basel). 2022 Nov 23;11(12):1692. doi: 10.3390/biology11121692 (PMC9774717; doi:10.3390/biology11121692)
Supplement: Supplementary file 1 [file biology-11-01692-s001.zip › biology-2017863-supplementary.pdf]

a)

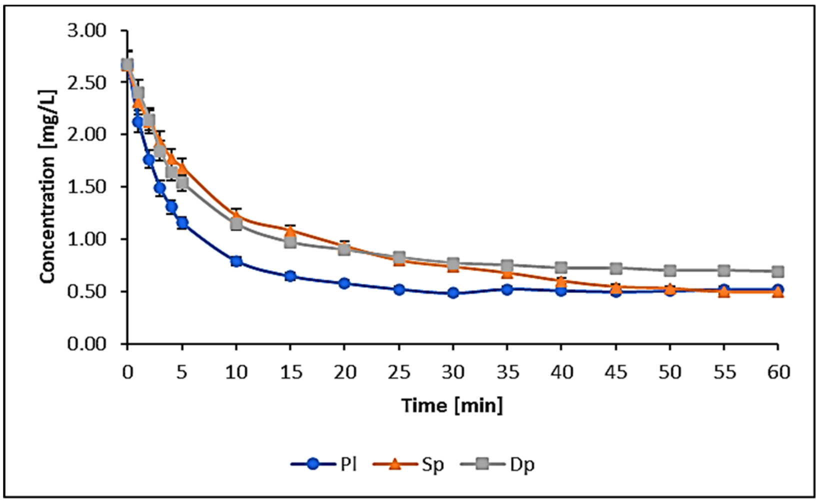
Concentration [mg/L]
3.00
2.50
2.00
1.50
1.00
0.50
0.00
0 5 10 15 20 25 30 35 40 45 50 55 60
Time [min]
Pl
Sp
Dp

b)

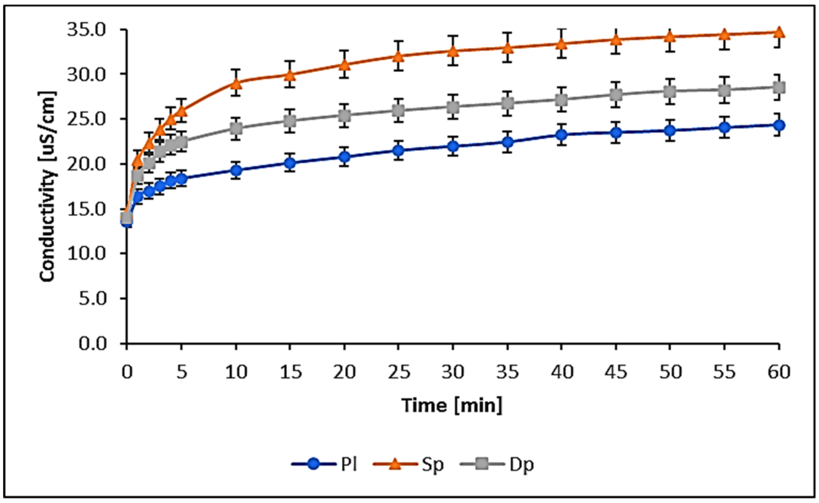
Conductivity [uS/cm]
35.0
30.0
25.0
20.0
15.0
10.0
5.0
0.0
0 5 10 15 20 25 30 35 40 45 50 55 60
Time [min]
Pl
Sp
Dp

c)

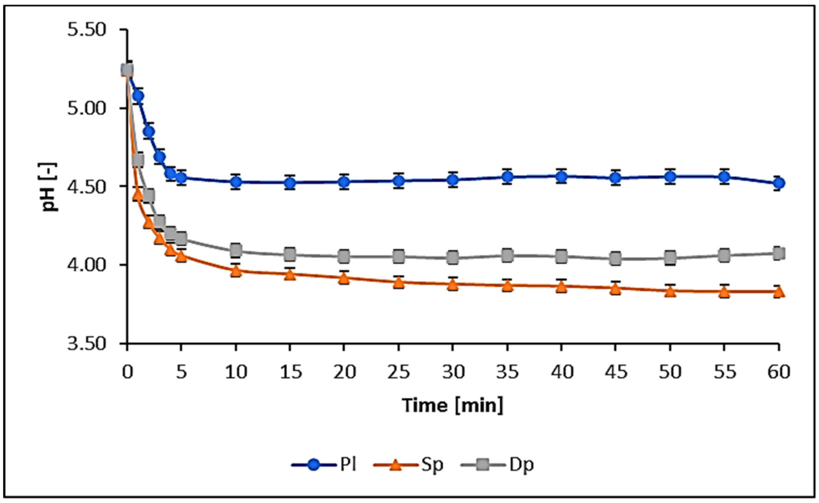

Figure S1. Changes in physicochemical parameters in Ni solution during the accumulation process on moss gametophytes: a) its concentration, b) conductivity, c) pH

a)

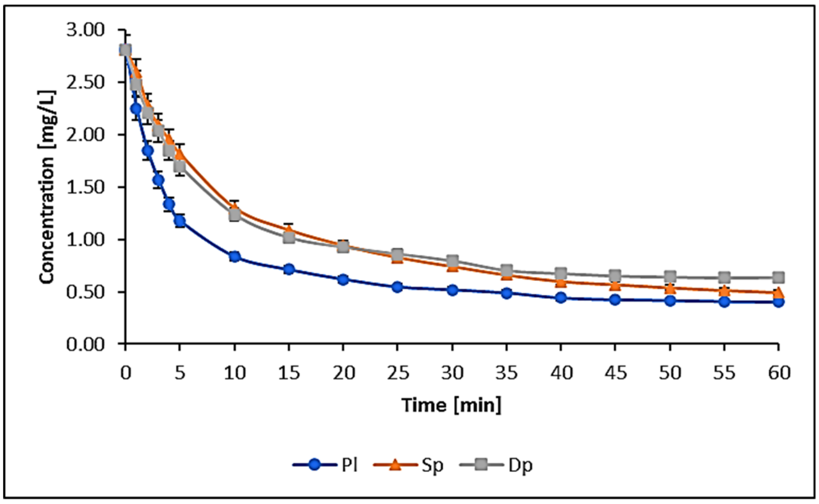

b)

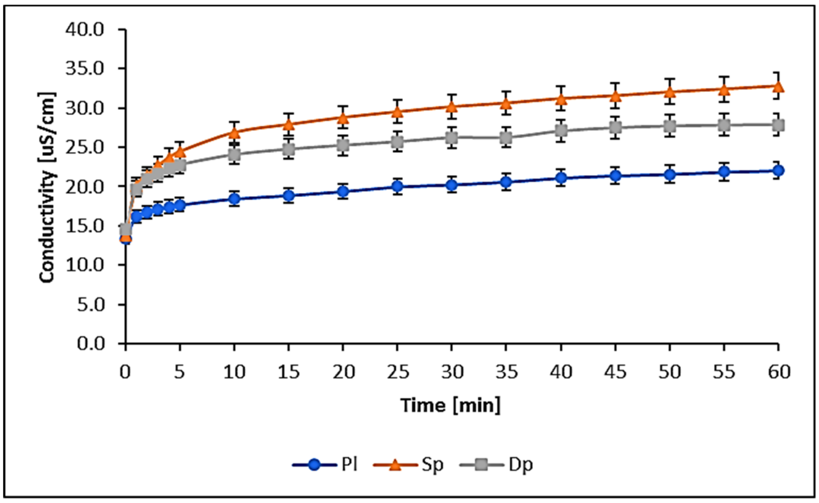

c)

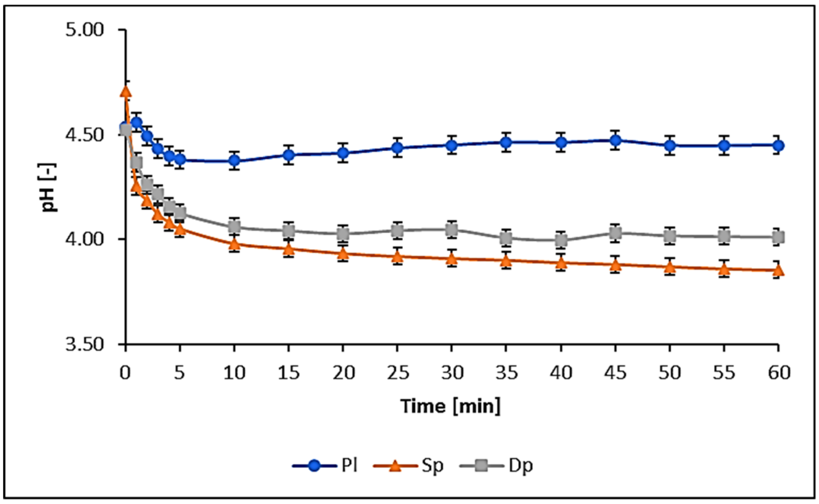

Figure S2. Changes in physicochemical parameters in Zn solution during the accumulation process on moss gametophytes: a) its concentration, b) conductivity, c) pH

a)

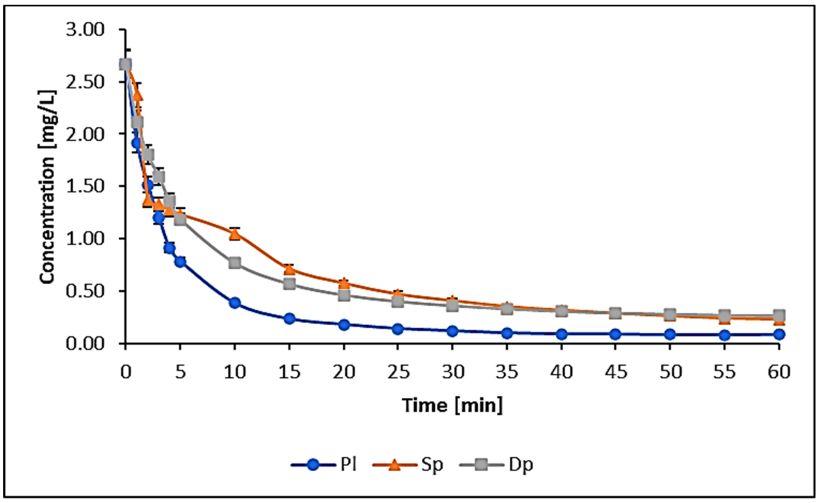

Concentration [mg/L]
Time [min]
Pl
Sp
Dp

b)

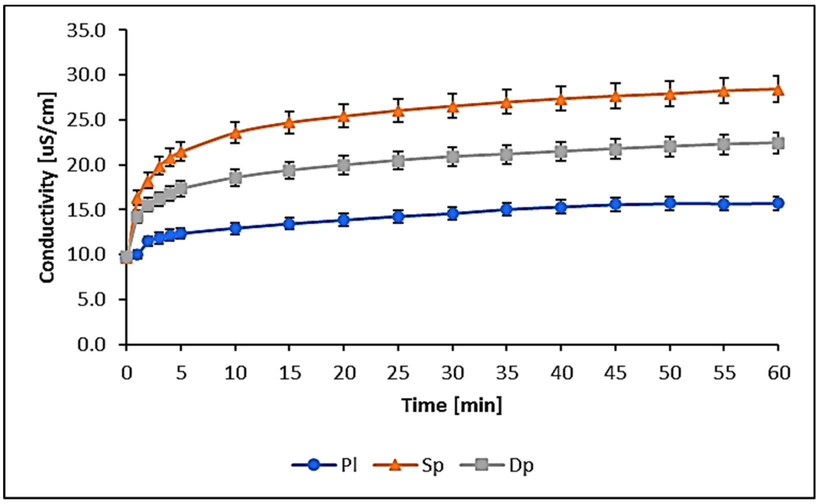

Conductivity [uS/cm]
Time [min]
Pl
Sp
Dp

c)

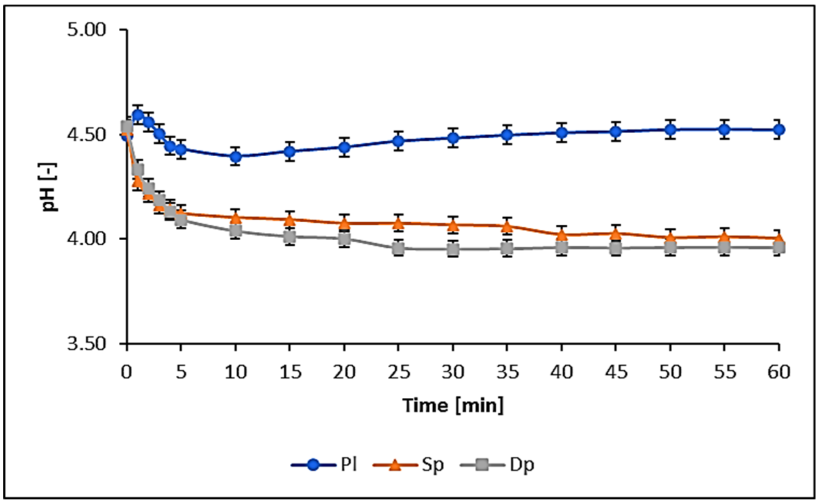

pH [-]
Time [min]
Pl
Sp
Dp

Figure S3. Changes in physicochemical parameters in Cd solution during the accumulation process on moss gametophytes: a) its concentration, b) conductivity, c) pH

a)

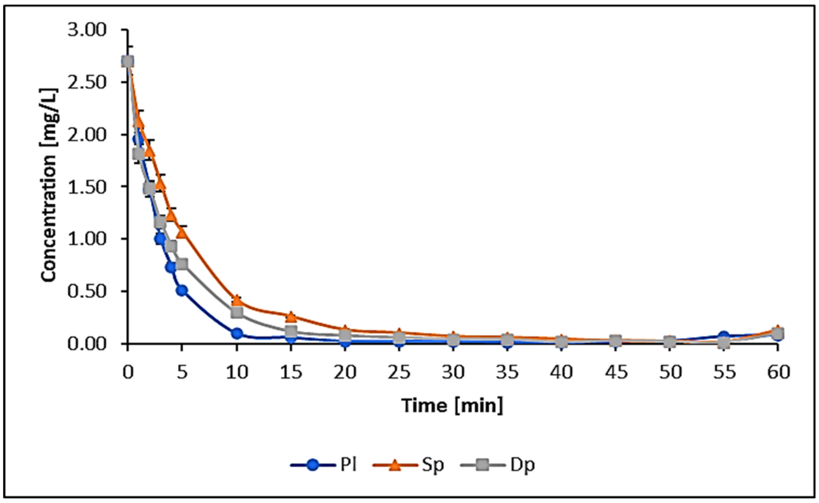

b)

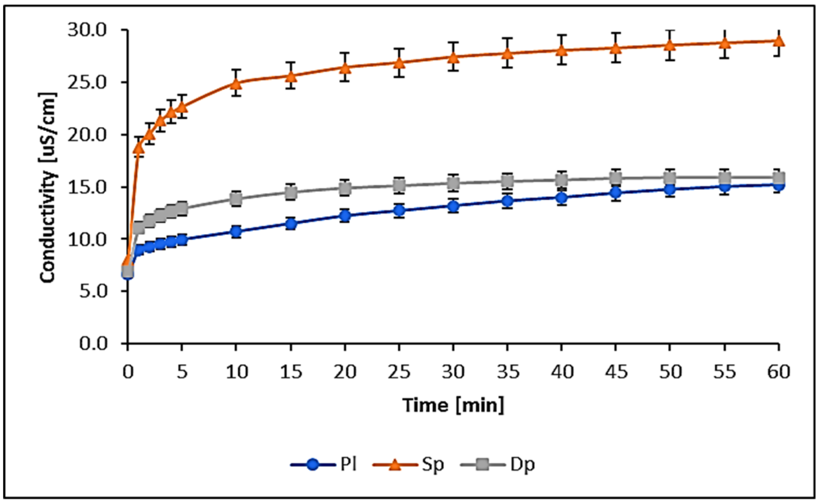

c)

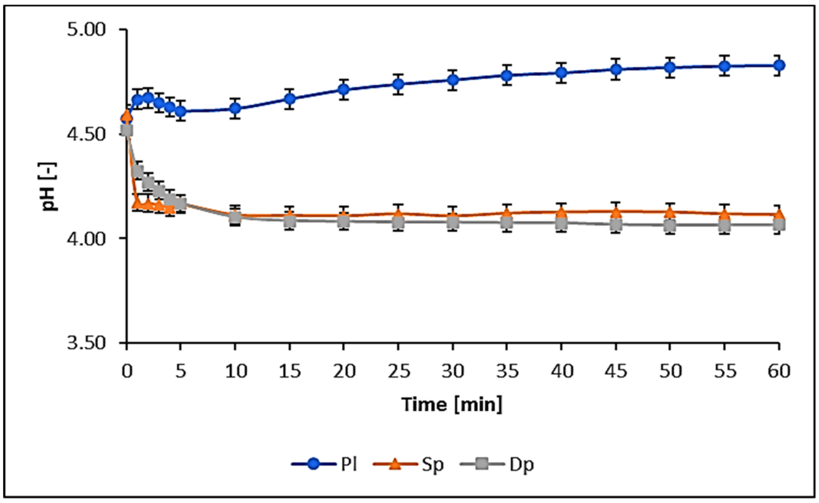

Figure S4. Changes in physicochemical parameters in Pb solution during the accumulation process on moss gametophytes: a) its concentration, b) conductivity, c) pH

Table S1. Concentrations of metals naturally accumulated in the mosses used for the experiments [mg/g d.w.]

Biomonitor	Ni	Cu	Zn	Cd	Pb
P. schreberi	< 0.001	< 0.001	0.04	< 0.0003	0.003
S. fallax	< 0.001	< 0.001	0.03	< 0.0003	< 0.002
D. polysetum	< 0.001	< 0.001	0.04	< 0.0003	< 0.002
